# A Proof of Concept Study of Function-Based Statistical Analysis of fNIRS Data: Syntax Comprehension in Children with Specific Language Impairment Compared to Typically-Developing Controls

**DOI:** 10.3389/fnbeh.2016.00108

**Published:** 2016-06-07

**Authors:** Guifang Fu, Nicholas J. A. Wan, Joseph M. Baker, James W. Montgomery, Julia L. Evans, Ronald B. Gillam

**Affiliations:** ^1^Department of Mathematics and Statistics, Utah State UniversityLogan, UT, USA; ^2^Department of Psychology, Utah State UniversityLogan, UT, USA; ^3^Center for Interdisciplinary Brain Sciences Research, Division of Brain Sciences, Department of Psychiatry and Behavioral Sciences, School of Medicine, Stanford UniversityStanford, CA, USA; ^4^Department of Communication Disorders, Ohio UniversityAthens, OH, USA; ^5^School of Behavioral and Brain Sciences, University of Texas at DallasRichardson, TX, USA; ^6^Department of Communicative Disorders and Deaf Education, Utah State UniversityLogan, UT, USA

**Keywords:** fNIRS, hemodynamic response curve, functional data analysis, specific language impairment, sentence comprehension

## Abstract

Functional near infrared spectroscopy (fNIRS) is a neuroimaging technology that enables investigators to indirectly monitor brain activity *in vivo* through relative changes in the concentration of oxygenated and deoxygenated hemoglobin. One of the key features of fNIRS is its superior temporal resolution, with dense measurements over very short periods of time (100 ms increments). Unfortunately, most statistical analysis approaches in the existing literature have not fully utilized the high temporal resolution of fNIRS. For example, many analysis procedures are based on linearity assumptions that only extract partial information, thereby neglecting the overall dynamic trends in fNIRS trajectories. The main goal of this article is to assess the ability of a functional data analysis (FDA) approach for detecting significant differences in hemodynamic responses recorded by fNIRS. Children with and without SLI wore two, 3 × 5 fNIRS caps situated over the bilateral parasylvian areas as they completed a language comprehension task. FDA was used to decompose the high dimensional hemodynamic curves into the mean function and a few eigenfunctions to represent the overall trend and variation structures over time. Compared to the most popular GLM, we did not assume any parametric structure and let the data speak for itself. This analysis identified significant differences between the case and control groups in the oxygenated hemodynamic mean trends in the bilateral *inferior frontal* and left *inferior posterior parietal* brain regions. We also detected significant group differences in the deoxygenated hemodynamic mean trends in the right *inferior posterior parietal cortex* and left *temporal parietal junction*. These findings, using dramatically different approaches, experimental designs, data sets, and foci, were consistent with several other reports, confirming group differences in the importance of these two areas for syntax comprehension. The proposed FDA was consistent with the temporal characteristics of fNIRS, thus providing an alternative methodology for fNIRS analyses.

## 1. Introduction

Functional near infrared spectroscopy (fNIRS) is a non-invasive method for measuring near-infrared light absorption through the skull, enabling researchers to speculate a close proxy to neural activation that results from relative changes of the cerebrovascular alterations in oxygenated and deoxygenated hemoglobin concentrations in cortical structures (Villringer and Dirnagl, 1994; Boas et al., 2014; Tak and Ye, 2014). Since light between 650 and 950 nm is weakly absorbed by biological chromophores (Hoge et al., 2005), the relatively deep penetration of NIR light makes it an effective research tool in neuro-imaging studies. Compared to other imaging technologies such as functional magnetic resonance imaging (fMRI) and positron emission tomography (PET), fNIRS has a few advantages such as low cost, high flexibility, portability, and the ability to accommodate young children and patients with psychological issues (Arenth et al., 2007; Ye et al., 2009). fNIRS offers superior temporal resolution with dense measurements over time and provides data for a wide range of functional contrasts such as oxygenated (Δ*HbO*), deoxygenated (Δ*HbD*), and total hemoglobin (Δ*HbT*) simultaneously as participants perform functional tasks in naturalistic environments (Kozel et al., 2009; Ye et al., 2009; Hall et al., 2013; Tak and Ye, 2014). Despite the extensive study of fNIRS data, little has been done to study the mean and variation trends of hemodynamic curves as individuals complete language processing tasks. Indeed, analysis approaches that truly utilize the superior temporal characteristics of fNIRS are rare in the existing literature. Even rarer are studies of concomitant behavioral and neural differences between children with specific language impairment (SLI) and typically developing control children as they complete language comprehension tasks.

In this article, we introduce a functional data analysis (FDA) methodology with a goal of addressing several challenging questions: (1) how to best utilize the superior temporal resolution of fNIRS; (2) how to model its hemodynamic trends for syntax-related stimuli; (3) how to connect light optodes with brain regions without anatomy information; (4) how to speculate the differences in brain activities between case and control in reaction to the same stimuli. FDA is a nonparametric data-driven statistical technique that does not make any parametric assumptions such as linearity or normality. Our main objective was to model the overall hemodynamic trends from a functional perspective as opposed to individual discrete points that are considered using existing analysis approaches. Although the modeling goal of FDA conforms to the temporal hemodynamic signals of the fNIRS context (Barati et al., 2013), it has seldom been applied in the fNIRS literature.

Tak and Ye (2014) reviewed currently existing statistical models in fNIRS data. The most well-known and widely used method was the GLM (Schroeter et al., 2004; Plichta et al., 2007), which has been integrated into numerous fNIRS analysis tools (Shimada and Hiraki, 2006; Koh et al., 2007; Abdelnour and Huppert, 2009; Huppert et al., 2009; Strangman et al., 2009; Ye et al., 2009; Custo et al., 2010; Penny et al., 2011). As a multivariate statistical model, GLM works well, but FDA differs in important ways. First, GLM is a traditional parametric model that assumes a linear combination structure. Assuming a parametric form would likely be misleading if the underlying data did not satisfy the main linear assumptions. Therefore, nonparametric modeling without any assumptions should be more flexible. Second, as a multivariate model, GLM does not utilize the time course of the data and hence can not capture the overall trends of the hemoglobin concentration in the dynamic or functional sense (Barati et al., 2013). Third, GLM does not provide a relevant hypothesis test approach to compare the differences in the overall hemodynamic trends between case and control groups due to its model structure restrictions.

Comparing which brain regions are significantly involved in a task performed by two groups requires formal hypothesis testing. Unfortunately, many of the current statistical approaches used to perform hypothesis tests for fNIRS data may not be optimal in the functional sense. Simple statistics such as *t*-test have been performed to statistically compare single-value differences between different groups (Aldrich et al., 1994; Germon et al., 1994, 1999; Young et al., 2000; Hoshi et al., 2001; Isobe et al., 2001; Kennan et al., 2002; Schroeter et al., 2002; Hoshi, 2003; Matsuo et al., 2003; Tachtsidis et al., 2004; Tsujimoto et al., 2004; Shibuya-Tayoshi et al., 2007; Kim et al., 2010). Multi-way ANOVA has also been employed in fNIRS studies (Fallgatter and Strik, 1998; Bartocci et al., 2000; Fallgatter and Strik, 2000; Herrmann et al., 2003; Hoshi, 2003; Suto et al., 2004; Folley and Park, 2005; Kameyama et al., 2006; Arenth et al., 2007; Irani et al., 2007). Although these methods were able to evaluate differences in hemoglobin observations, information was lost because only partial measurements were considered. Using FDA to compare the overall temporal mean and variation trends of hemodynamic response functions rather than simply defining a magnitude may be more informative and robust, especially in a context in which optical signal attenuation or motion artifacts cause noise (Ye et al., 2009).

When repeated measurements are recorded over a dense grid of time points, often by machine, they are typically termed as functional or longitudinal data, with one observed curve per subject. Formally, FDA models each hemodynamic response curve as a continuum function over time, thus capturing the overall dynamic trajectories of the function over time, even though the measurements are collected discretely (Ramsay and Silverman, 2002; Ferraty and Vieu, 2006; Ramsay, 2006; Barati et al., 2013). Although some experimental errors are generally unavoidable, nonparametric kernel smoothing captures the underlying mean function and hence greatly reduces the effects of noise. The functional principal component analysis (FPCA) based on the Karhunen-Loeve theorems decomposes the high dimensional auto-covariance matrix extracted from fNIRS data to a few important orthogonal eigenfunctions. The first few eigenfunctions explaining the majority of variation are likely induced by cognitive related tasks, with the remaining eigenfunctions explaining only a very small percentage of variation that may be caused by nuisance factors such as breathing, vasomotor, measurement error, movement artifacts, and other unaccounted activities (Akgül et al., 2006). To perform comprehensive comparisons on the hemodynamic curves between case and control groups, we tested the equality of mean functions and eigenfunctions and eigenvalues of the auto-covariance functions using two-sample FPCA approaches. Bootstrap sampling was used to determine the threshold of the significance of the tests because the distributions of the test statistics were unknown (Benko et al., 2009). Importantly, FDA is inherently nonparametric and does not assume any parametric structure or distributions within the hemodynamic curve data.

Some researchers have investigated the functional relationship between fNIRS and fMRI and their correlation over time (Mandeville et al., 1999; Siegel et al., 2003; Fujiwara et al., 2004; Okamoto et al., 2004; Steinbrink et al., 2006). Although many common properties exist between fNIRS and fMRI, functional curve based modeling, which is mature in fMRI research (Grodzinsky, 2000; Ben Schachar et al., 2003; Müller et al., 2003; Ben-Shachar et al., 2004; Weismer et al., 2005; Binder et al., 2009; Seghier et al., 2010; Seghier, 2013), has rarely been used for fNIRS stand-alone experiments. The progress achieved in fMRI analyses paves the way for improvements on fNIRS approaches. We believe that the FDA approach could promote breakthroughs in fNIRS research, similar to the way it did for fMRI.

To test the potential of FDA to analyze fNIRS data, we used fNIRS to asssess differences in neural activation between children (case: children with SLI; control: age-matched, typically-developing children) as they engaged a language comprehension task that is known to favor the children in the control group. SLI is a developmental language disorder of unknown origin that is characterized by significant deficits in the acquisition and use of spoken and written language in the absence of hearing, intellectual, emotional, or acquired neurological impairments (Bishop, 2014; Leonard, 2014). This disorder affects approximately 7% of the school-age population (Tomblin et al., 1997). If FDA is a promising statistical approach for fNIRS, it should reveal group differences in parasylvian (language related) neural regions as children perform the task.

## 2. Materials and methods

### 2.1. Participants

Thirty children (15 children with SLI and 15 age-matched, typically developing control children) between the ages of 8 and 12 participated in the study. There were eight males in each group. The children in the SLI group met the standard classification criteria of performance on multiple language measures that was one or more standard deviations below the mean. The typically-developing controls performed above one standard deviation from the mean on multiple language measures. All the children in both groups were right-handed, monolingual English speakers. All the children in the SLI group were receiving special education services in the public schools. In addition, we provided independent testing to insure that the children in the SLI group met our identification criteria.

### 2.2. Sentence comprehension task

The children completed a language comprehension task in which they listened to a sentence and then selected a picture (from three choices) that depicted the agent (actor) in the sentence. There were 60 total sentences with 15 sentences representing each of four sentence types: subject-verb-object (“The ring had moved the square behind the very bright cold bed”), subject relatives (“The watch that had hugged the truck behind the kite was bright”), passives (“The shoe was hugged by the clock under the very cold box”), and object relatives (“The book that the shirt had hugged under the kite was new”). The sentences were controlled for length, vocabulary complexity, and vocabulary imageability (Montgomery et al., 2015). Similar to Dick et al. (2004), noun animacy and noun affordance cues were removed, making the sentences semantically implausible. This was done so that the children's decisions about the agent of the sentence would be based primarily on syntactic knowledge or word order rather than semantic plausibility. Children saw three pictures on a computer screen as they listened to each sentence. They were asked to point to the picture of the agent of the sentence (the thing doing the action) as quickly as possible after hearing each sentence. All children completed eight training items before fNIRS scanning began. See Montgomery et al. (2015) for a complete description of the stimuli.

### 2.3. Functional near infrared spectroscopy procedures

**Figure 1:**
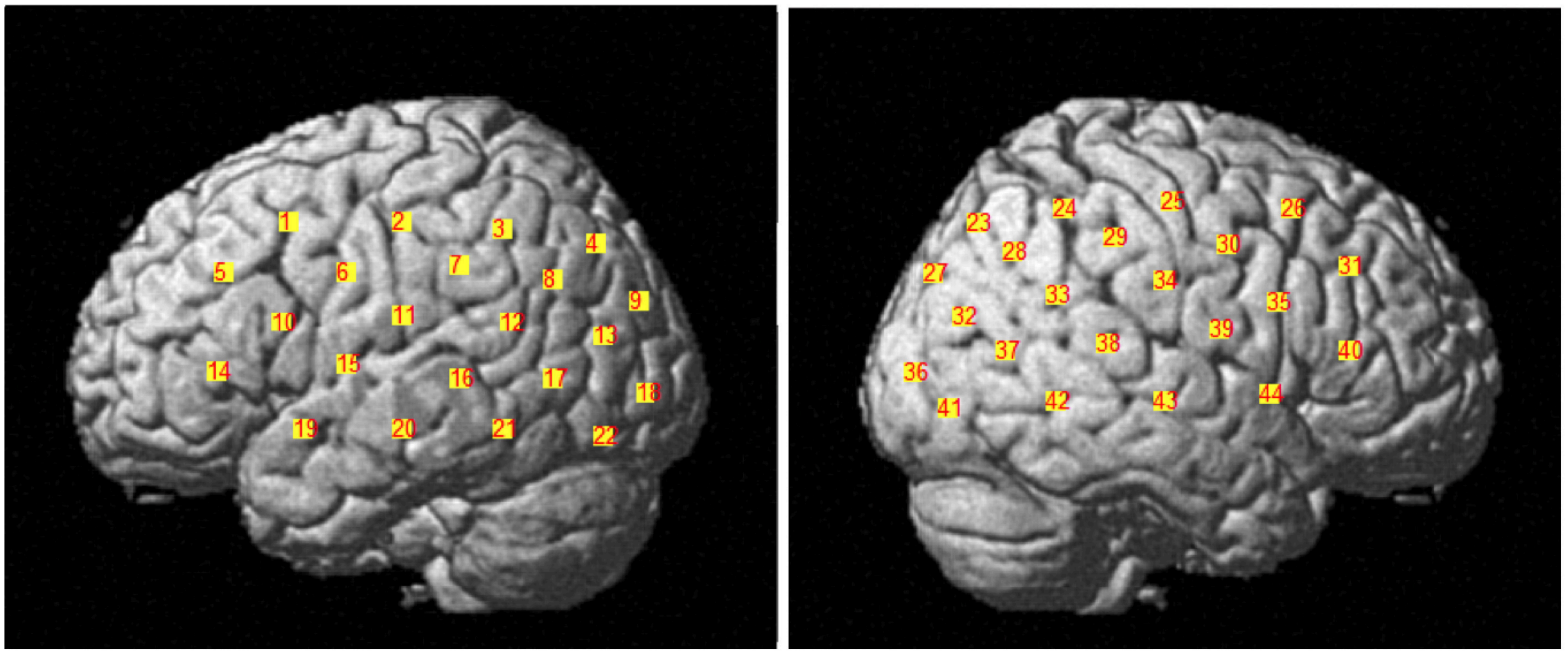
**Display of the 44 channels divided across two 3 × 5 probe caps**. The channels 1–22 belong to the left brain hemisphere and the channels 23–44 belong to the right brain hemisphere.

Data was collected with the Hitachi ETG-4000 (Hitachi Medical Co., Japan) with 44 channels divided across two 3 × 5 probe caps. The channels were determined by bilateral placement of the optode caps such that the middle detector in the lowest row of optodes was placed over T3 or T4. The measurement patches covered the majority of the right and left parasylvian regions including *inferior frontal cortex, inferior parietal lobule* (including the *temporal parietal junction* and *inferior posterior parietal cortex*), and *superolateral temporal cortex*. The channel locations are depicted in Figure 1.

The fNIRS scan began with a 30 s rest period in which children were instructed to focus on a “+” in the middle of the computer screen and to “relax” their mind. After the first rest period, children listened to 60, 12-word sentences representing four different syntax types (15 subject-verb-object sentences, 15 subject relative clause sentences, 15 passive sentences, and 15 object relative clause sentences). E-prime software was used to present the stimuli in a pseudo-random order and to record the accuracy and speed of the children's responses. The sentences were presented in three blocks of 20 items, presented in a psudorandom order, with each item being separated by a jittered rest interval that varied between 2 and 6 s. Each block was separated by a 25 s rest period. The stimuli onsets for each participant were consistently predefined and each participant was given 8 s to think and respond.

**Figure 2:**
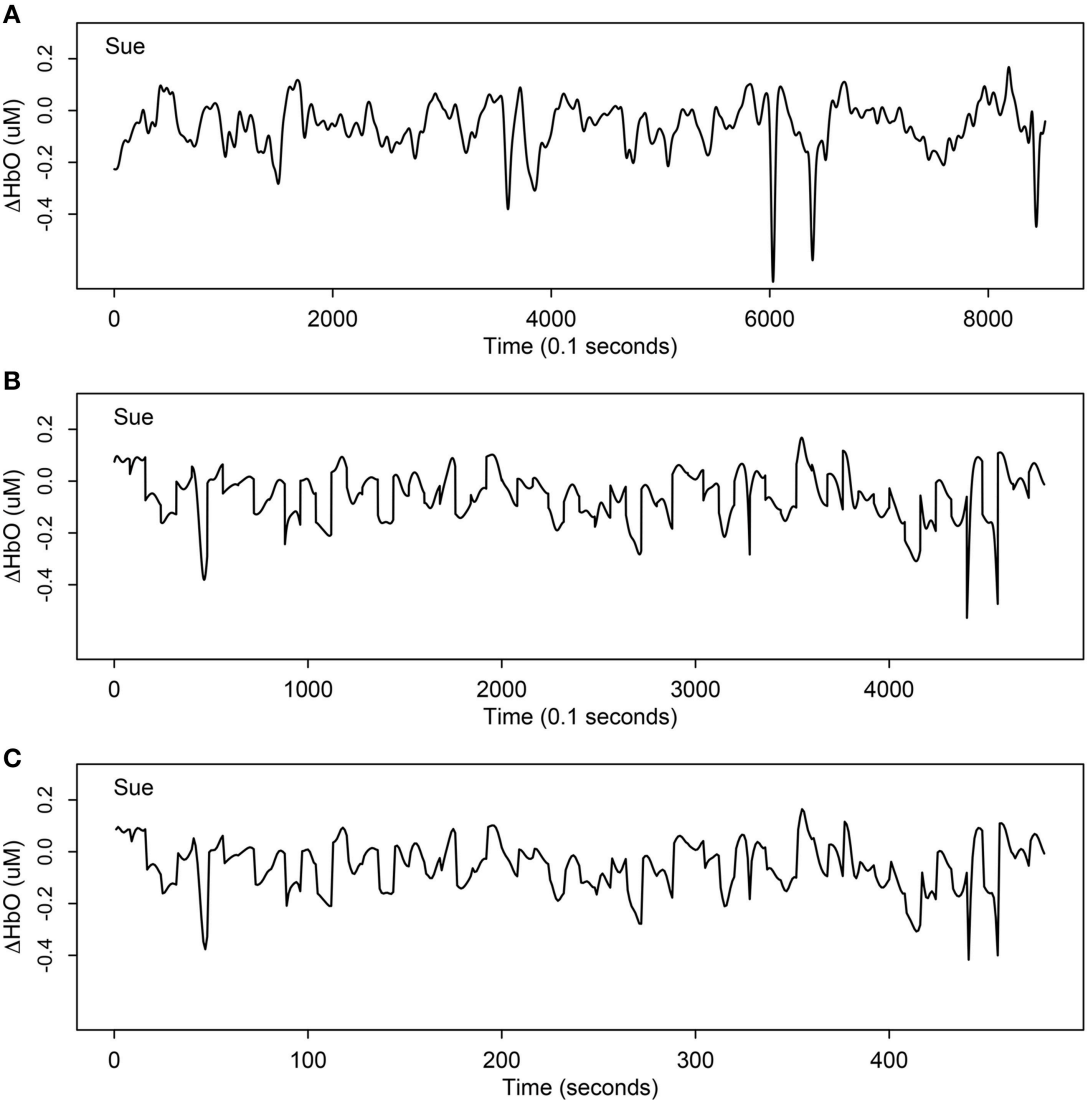
**One example of Δ***HbO*** time series at channel 31 (mainly overlapped in the right ***inferior frontal cortex***) for Sue, a child participant with specific language impairment**. **(A)** The original time series of Δ*HbO* with length 8521; **(B)** the extracted stimulus-relevant Δ*HbO* under 60 target stimuli instants with length 4800; **(C)** the average version of **(B)** with length 480. By averaging the 10 measurements of each second, the curve maintains similar signal but only using 1/10 of original length.

Throughout the fNIRS scan, near-infrared light from the source optodes travels approximately 1–1.5 cm into the cortex where it is absorbed by oxygen molecules attached to hemoglobin in the blood in the brain (Dehghani and Delpy, 2000). The amount of light that is not absorbed is measured by the detecting optodes. The relative changes in the concentration of oxygenated hemoglobin (Δ*HbO*), deoxygenated hemoglobin (Δ*HbD*), and total hemoglobin (Δ*HbT*) were estimated according to changes in the optical properties of the light using the Beers-Lambert conversion (see Plichta et al., 2007 for a detailed description). A length of 8521 and a frequency of 10 Hz time series was collected within a duration of 851 sec for each channel of each participant. Figure 2A displays one example of original Δ*HbO* time series at channel 31 (mainly overlapped in the right *inferior frontal cortex*) for a child in the SLI group.

### 2.4. Data preprocessing

There were a total of 3960 individual time series collected from three hemoglobin categories (Δ*HbO*, Δ*HbD*, and Δ*HbT*), 44 channels, and 30 participants (15 cases and 15 controls). Each time series contained 8521 measurement units consisting of 4800 intermittent task measurement units and 3721 rest measurement units. The active periods represented 15 stimuli segments for each of the four syntax types. The following preprocessing steps were designed to extract the most important information from such a large amount of data.

The first step of data preprocessing was to group channels based on regions of interest (ROIs). The global alignments of the channel positions between individuals were difficult because fNIRS has the shortcoming of weak spatial anatomical representation. The ROIs for the current project were derived a priori based on previous findings in both the fMRI and fNIRS literature demonstrating changes in cortical activation during language processing tasks. Four areas within the parasylvian region, *inferior frontal cortex* (*Broca's* area), *superolateral temporal cortex*, the *temporal parietal junction* and *posterior inferior parietal cortex* (*Angular Gyrus*) are frequently implicated in verbal tasks (Rossi et al., 2012; Scherer et al., 2012; Petrides, 2013). A Polemus system was used for 3D digitization of head size and optode location following testing. This provided standardized Montreal Neurological Institute coordinates and anatomical labels that related to each participant individually. We determined the corresponding channel for each monitored brain region based on the largest percentage of overlapping rate between the channel and the brain ROI for each participant.

The second step of data preprocessing was to extract only stimulus-related active units from the original time series and focus only on the segments associated with cognitive activity during the target stimulus comprehension tasks. There were 60 such windowed segments, each lasting 8 sec (corresponding to 80 units), and hence, a total 4800 units were extracted. As an example, Figure 2B displays the stimulus- relevant Δ*HbO* extracted from the original time series at channel 31 (mainly overlapping the right *inferior frontal cortex*) for a child (Sue) in the SLI group. This process was repeated for all individuals.

Since the observations were collected very densely, we used the average of the 10 units per second as the modeling target, illustrated in Figure 2C. Comparing Figure 2B and Figure 2C, notice that the two signals look almost the same, except Figure 2B has length 4800 but Figure 2C is only of length 480 (1/10 of original length). If there were any differences caused by averaging the 10 dense units per second (Figure 2C), it would be smoother and would capture the trend even better by removing more noise or errors from averaging.

**Figure 3:**
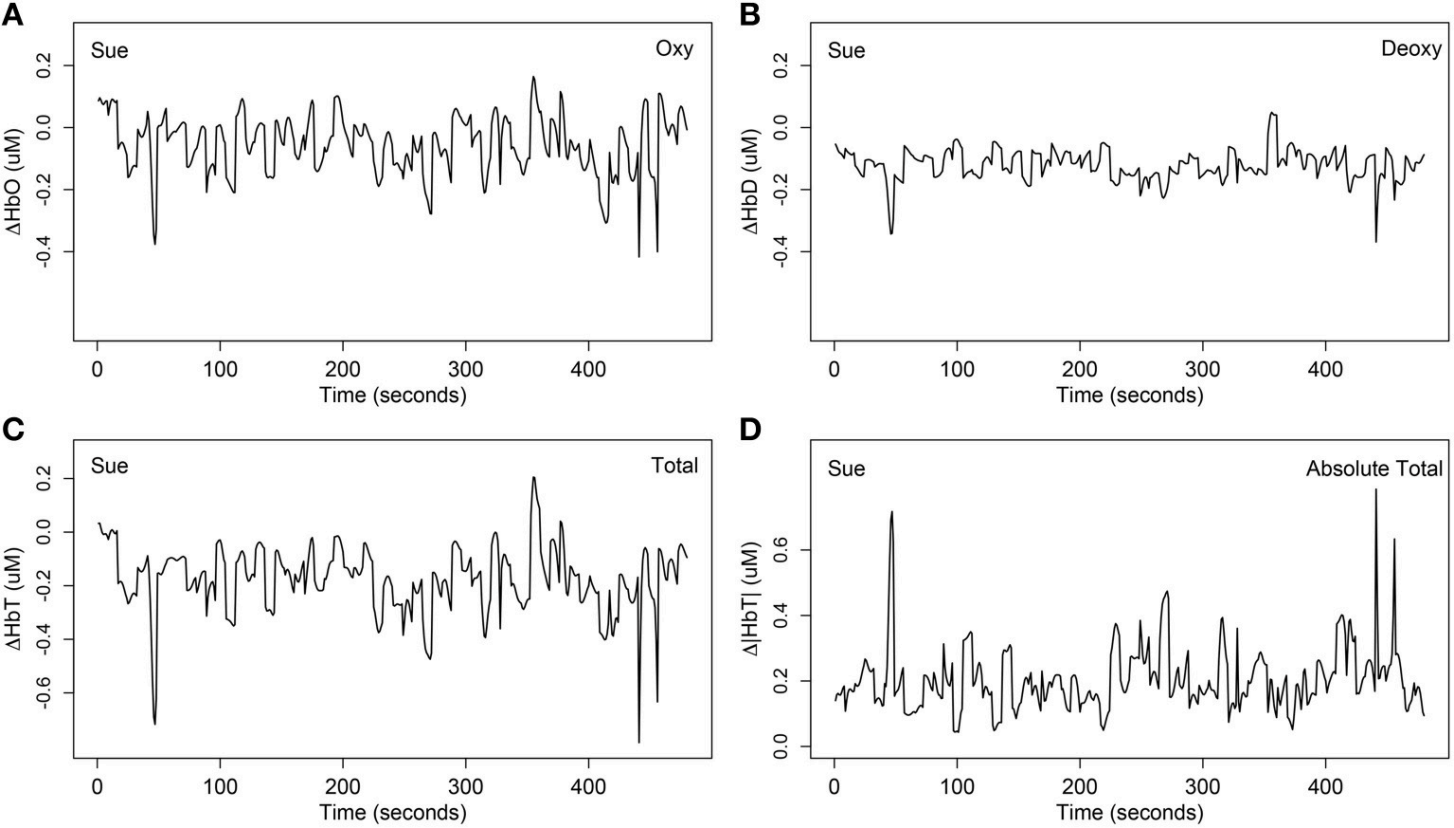
**One example of the four different stimulus-relevant hemoglobin categories at channel 31 (mainly overlapped in the right ***inferior frontal cortex***) for Sue**. **(A)** The stimulus-relevant oxygenated hemodynamic curve Δ*HbO*; **(B)** the stimulus-relevant deoxygenated hemodynamic curve Δ*HbD*; **(C)** the stimulus-relevant total hemodynamic curve Δ*HbT*; **(D)** the stimulus-relevant absolute total hemodynamic curve Δ|*HbT*|. Δ*HbT* was computed by summing Δ*HbO* and Δ*HbD*. Absolute total Δ|*HbT*| was computed by summing the absolute value of Δ*HbO* and Δ*HbD*.

The third step of data preprocessing related to selecting the hemoglobin categories. It is not clear whether neuronal activation is best represented by Δ*HbO*, Δ*HbD*, or Δ*HbT*. Researchers may expect the deoxygenated hemoglobin to show opposite trends to that of Δ*HbO* because the Δ*HbO* and Δ*HbD* often complement each other (Cui et al., 2010). However, comparing Figure 3A with Figure 3B for one example of the same channel for the same person, note that the deoxygenated hemodynamic trends are flatter than the oxygenated hemodynamic trends, and there does not appear to be opposite trends in most time segments. This suggests that the oxygenated hemoglobin contains a more rubust signal than the deoxygenated hemoglobin. In this article, we mainly focused on modeling Δ*HbO* and Δ*HbD* because the results of Δ*HbT* (the sum of Δ*HbO* and Δ*HbD*) were highly correlated with the other two.

**Figure 4:**
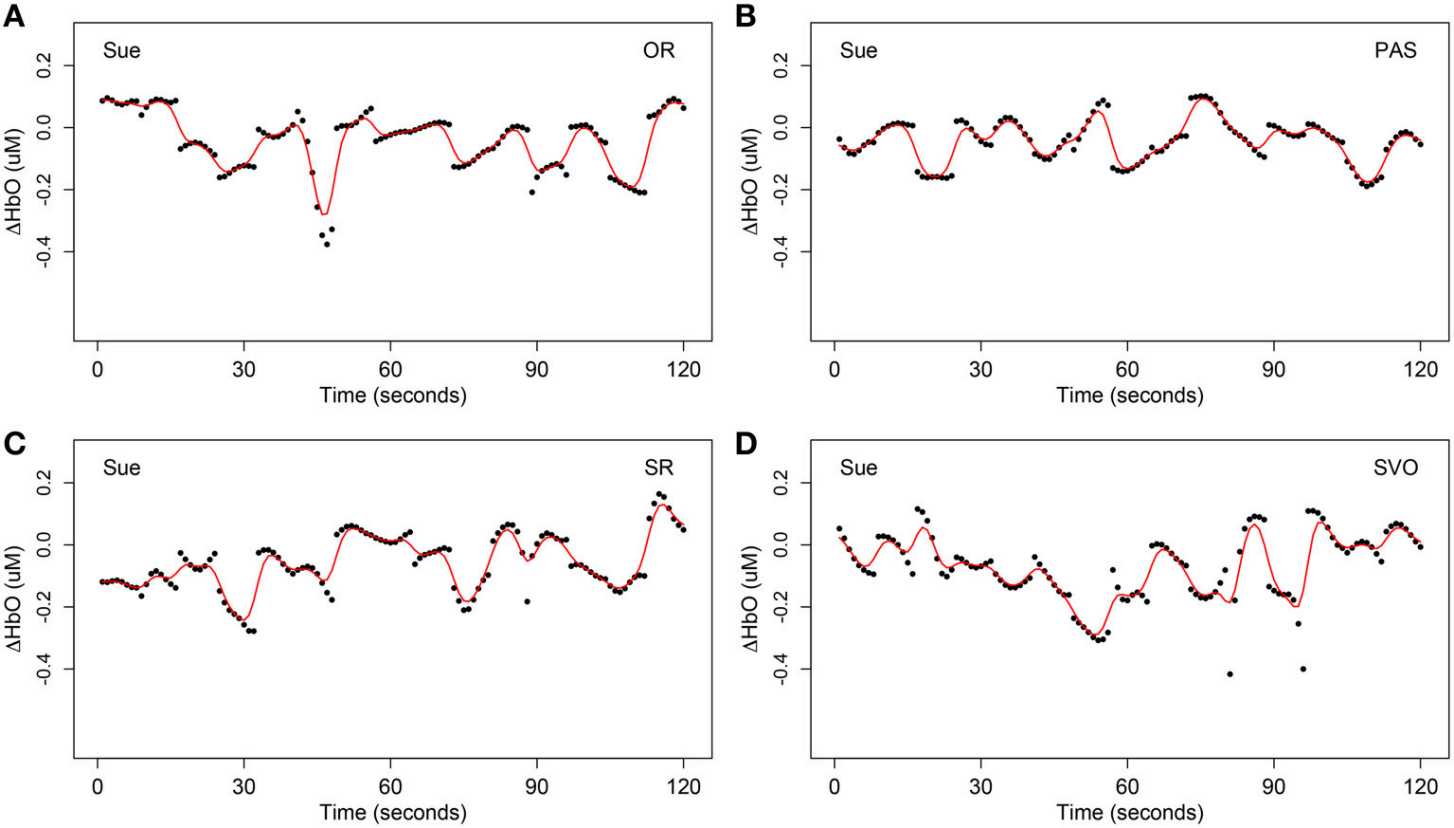
**One example of extracted syntax-relevant Δ***HbO*** at channel 31 (mainly overlapped in the right ***inferior frontal cortex***) for Sue under four syntax types respectively, each with 15 target stimulus questions**. The black dots are the original observation *Y*_*ikc*_ of oxygenated hemoglobin and the red curves are smoothing hemodynamic trajectories *X*_*ic*_(*t*) estimated by nonparametric kernel smoother from model Equation (1). **(A)** Object relative clause sentences (OR); **(B)** passive sentences (PAS); **(C)** subject relative clause sentences (SR); **(D)** subject-verb-object sentences (SVO).

The forth step of data preprocessing involved extracting the syntax-relevant time course by locating the time onsets of the 15 questions for each syntax type. This yielded four different time courses, each with 120 units. As an example, Figures 3, 4 display one example of the four syntax-relevant Δ*HbO* hemodynamic curves extracted from the original time series at channel 31 (mainly overlapped in the right *inferior frontal cortex*) for a child in the SLI group named Sue.

Factors such as breathing, vasomotor response, measurement error, movement artifacts, and other unaccounted activities (Akgül et al., 2006), may cause noise in fNIRS data. These four preprocessing steps enabled us to extract the most important signals and remove unavoidable confounding factors. Comparing Figure 2A with Figure 4, notice that it is harder to recognize patterns from Figure 2A due to many complex and sharp fluctuations and strands. On the contrary, the patterns are smoother and clearer in Figure 4. After these preprocessing steps, our data were ready for the statistical models and hypothesis tests.

### 2.5. Functional data analysis structure

Let *Y*_*ikc*_, *i* = 1, …, *n*; *k* = 1, …, *T*; *c* = 1, 2, denote the relative changes in the concentrations of oxygenated or deoxygenated hemoglobin of the *ith* subject measured at discrete time point *t*_*k*_ for the *cth* group. Here *c* = 1 denotes the case group and *c* = 2 denotes the control group, *n* is the number of subjects per group, and *T* is the total time points measured for each subject. These observed densely collected curves with noise can be modeled as independent realizations of a stochastic process with smooth trajectories.

Let *X*_1*c*_(*t*), …, *X*_*nc*_(*t*) denote random smooth trajectories of the underlying stochastic process in *L*^2^(T), *t* ∈ T, where T is the time interval. Then we can reconstruct the smooth functions $X_{i}^{\prime }s$ from the original densely collected noisy observations $Y_{i}^{\prime }s$ as (Müller, 2008)

(1) $$\begin{array}{l} Y_{ikc}=X_{ic}\left(t\right)+\varepsilon_{ikc},\;i=1,\ldots ,n;\;c=1,2;\;k=1,\ldots ,T;\\ \;t\in T, \end{array}$$

where ε_*ikc*_ are the experimental errors and assumed to be independent, with *E*(ε_*ikc*_) = 0 and $Var\left(\varepsilon_{ikc}\right)=\sigma_{kc}^{2}$.

For each group *c*, the mean function of *X*_*ic*_(*t*) is μ_*c*_(*t*) = *E*(*X*_*ic*_(*t*)) and auto-covariance function of *X*_*ic*_(*t*) is

$$\begin{array}{l} G_{c}\left(s,t\right)=cov\left\{X_{ic}\left(s\right),X_{ic}\left(t\right)\right\}=E\left\{\left[X_{ic}\left(s\right)-\mu_{c}\left(s\right)\right]\left[X_{ic}\left(t\right)-\mu_{c}\left(t\right)\right]\right\}, \end{array}$$

for *s, t* ∈ T. Here μ_*c*_(*t*) is interpreted as the mean function of oxygenated or deoxygenated hemodynamic curves for group *c*. Throughout this paper, it is assumed that μ_*c*_(*t*) is a smooth function of *t*, and *G*_*c*_(*s, t*) is a positive definite and bivariate smooth function of *s* and *t*, for *s, t* ∈ T. The “smooth” refers to twice continuously differentiable. The idea of model Equation (1) is that the observed noisy curve over time is described by an underlying smooth function plus noise.

In order to model the auto-covariance function, functional PCA interprets *G*_*c*_(*s, t*) as the kernel of a linear integral operator on the space *L*_2_(T) of square-integrable functions on T, mapping *f* ∈ *L*_2_(T) to *A*_*G*_*c*__*f* ∈ *L*_2_(T) defined by

(2) $$\begin{array}{r} (A_{G_{c}}f)(t)=\int_{T}f(s)G_{c}(s,t)ds. \end{array}$$

An eigenfunction *v* of the auto-covariance operator *A*_*G*_*c*__ is a solution of the equation (*A*_*G*_*c*__*v*)(*t*) = λ*v*(*t*), with eigenvalue λ. For each *c*, we assume that the operator *A*_*G*_*c*__ has a sequence of smooth orthonormal eigenfunctions *v*_*lc*_ satisfying $\int_{T}v_{kc}(t)v_{lc}(t)dt=\delta_{kl}$ (here δ_*kl*_ is the Kronecker symbol), with ordered eigenvalues λ_1*c*_ ≥ λ_2*c*_ ≥ … ≥ 0. By Mercer's Theorem, applying a spectral decomposition on the function *G*_*c*_ yields

(3) $$\begin{array}{l} G_{c}\left(s,t\right)=\overset{\infty }{\underset{l=1}{\sum }}\lambda_{lc}v_{lc}\left(s\right)v_{lc}\left(t\right). \end{array}$$

Since the eigenfunctions *v*_*lc*_'s form a complete orthonormal sequence on *L*_2_(T), the generalized Fourier expansion (*Karhunen* − *Loeve* Theorem (Karhunen, 1946) or functional principal component expansion) on *X*_*ic*_ yields

(4) $$\begin{array}{l} X_{ic}\left(t\right)=\mu_{c}\left(t\right)+\overset{\infty }{\underset{l=1}{\sum }}\zeta_{ilc}v_{lc}\left(t\right),c=1,2, \end{array}$$

where the sum is defined in the sense of *L*_2_ convergence and

(5) $$\begin{array}{l} \zeta_{ilc}=<X_{ic}-\mu_{c},v_{lc}>=\int_{T}(X_{ic}(t)-\mu_{c}(t))v_{lc}(t)dt \end{array}$$

are uncorrelated random variables with *E*(ζ_*ilc*_) = 0, and *var*(ζ_*ilc*_) = λ_*lc*_, subject to the *L*_2_ convergence, i.e.,

$$\Sigma_{l}\lambda_{lc}=E\left(||X_{ic}-\mu_{c}|{|}^{2}\right)=\int G_{c}\left(t,t\right)dt<\infty .$$

ζ_*lc*_ are frequently referred to as the *lth* functional principal component score or the *lth* dominant modes of random effects.

By way of Equation (4), the dynamic trends of random function *X*_*ic*_(*t*) can be modeled by the mean trend function μ_*c*_(*t*), the eigenfunction *v*_*lc*_, and the distribution of functional principal component scores ζ_*ilc*_. The first *L* principal components were used to approximate Equation (4) to capture the most important variations, remove the noise effects, and estimate the main signals of the trajectories of *X*_*c*_(*t*) effectively (Ramsay and Silverman, 2002).

### 2.6. Parameter estimates

Using the observed data set D = {*Y*_*ikc*_, *i* = 1, …, *n*; *k* = 1, …, *T*; *c* = 1, 2}, we were able to estimate all unknown parameters ${\hat{\mu }}_{c}\left(t\right)$, Ĝ_*c*_(*s, t*), and ${\hat{\sigma }}_{kc}^{2}$ from Equations (1) and (5). The smooth function *X*_*ic*_(*t*_*k*_) and ${\hat{\sigma }}_{kc}^{2}$ of each discrete noisy observation (*t*_*ik*_, *Y*_*ikc*_) were estimated by model Equation (1) via nonparametric kernel smoothing. Then the unbiased estimator of μ_*c*_(*t*) was easily obtained from the sample mean of *X*_*ic*_(*t*).

Once the estimator ${\hat{\mu }}_{c}\left(t\right)$ was obtained, we computed the sample estimate of auto-covariance matrix by

$${Ĝ}_{c}\left(t,s\right)=n^{-1}\Sigma_{i=1}^{n}\left\{X_{ic}\left(s\right)-{\hat{\mu }}_{c}\left(s\right)\right\}\left\{X_{ic}\left(t\right)-{\hat{\mu }}_{c}\left(t\right)\right\}.$$

The estimate of eigenfunctions were obtained by the corresponding spectral decomposition on Ĝ_*c*_(*s, t*). To be more specific, ${\hat{\lambda }}_{qc}$ are eigenvalues of Ĝ_*c*_, given by

$$\int_{T}{\hat{G}}_{c}(s,t){\hat{v}}_{lc}(s)ds={\hat{\lambda }}_{lc}{\hat{v}}_{lc}(t).$$

And ${\hat{v}}_{lc}$ is the eigenfunction corresponding to ${\hat{\lambda }}_{lc}$, satisfying $\int_{T}{\hat{v}}_{lc}^{2}(t)dt=1$ and $\int_{T}{\hat{v}}_{kc}{\hat{v}}_{lc}(t)dt=0$ if *k* ≠ *l*. The signs of ${\hat{v}}_{lc}$ were not uniquely determined. In order to ensure the closeness of ${\hat{v}}_{lc}$ from two groups of *c* = 1, 2, we allowed the signs of ${\hat{v}}_{lc}$ to be chosen arbitrarily as long as $<{\hat{v}}_{l1},\;{\hat{v}}_{l2}>\;\geq 0$ for *l* = 1, …, *L*.

Ĝ_*c*_ also presents an empirical version of the expansion (Equation 3)

(6) $$\begin{array}{l} {Ĝ}_{c}\left(s,t\right)=\overset{L}{\underset{l=1}{\sum }}I\left({\hat{\lambda }}_{lc}>0\right){\hat{\lambda }}_{lc}{\hat{v}}_{lc}\left(s\right){\hat{v}}_{lc}\left(t\right), \end{array}$$

where *I* is the indicator function used to only keep the terms with positive eigenvalues. From the percentage of variation explained by the first few eigenfunctions, the first *L* largest eigenvalues ${\hat{\lambda }}_{1c},\ldots ,{\hat{\lambda }}_{Lc}$ were chosen. The positive definiteness of the estimated auto-covariance matrix Ĝ_*c*_(*s, t*) was not always guaranteed, which might be a problem in practical applications. Once ${\hat{\lambda }}_{lc}$ and ${\hat{v}}_{lc}$ were obtained, we checked whether or not ${\hat{\lambda }}_{lc}>0$ (Müller, 2008). If ${\hat{\lambda }}_{lc}$ was negative, then we dropped this negative eigenvalue and its corresponding eigenfunction, and reconstituted the estimate from remaining eigenvalues and eigenfunction estimates.

Once eigenvalues ${\hat{\lambda }}_{1c}\geq \ldots \geq {\hat{\lambda }}_{Lc}$ and orthonormal eigenfunctions ${\hat{v}}_{1},\ldots ,{\hat{v}}_{L}$ were obtained, the fitting of individual trajectories required estimation of functional principal component scores. By the discretization on the Equation (5), plugging ${\hat{\mu }}_{c}$ and ${\hat{v}}_{lc}$ into a Riemann sum approximation of the integral, we have

(7) $$\begin{array}{l} {\hat{\zeta }}_{ilc}=\Sigma_{k=1}^{T}\left(X_{ic}\left(t_{k}\right)-\hat{\mu }\left(t_{k}\right)\right){\hat{v}}_{lc}\left(t_{k}\right)\left(t_{k}-t_{k-1}\right), \end{array}$$

setting *t*_0_ = 0 (Müller, 2008). We assured that $n^{-1}\Sigma {\hat{\zeta }}_{ilc}=0$, $n^{-1}\Sigma {\hat{\zeta }}_{ilc}{\hat{\zeta }}_{iwc}=0$ for *l* ≠ *w*; *l, w* = 1, …, *L*, and $n^{-1}\Sigma {\hat{\zeta }}_{ilc}^{2}={\hat{\lambda }}_{lc}$. This approximation method by sum worked well because our observations were collected densely and consistently for all subjects without missing values.

### 2.7. Nonparametric kernel smoothing

The nonparametric regression kernel smoothing was a traditional approach to capture the curve trends without making assumptions about the error distributions. The goal of smoothing was to model the underlying function by estimating *X*(*t*) = *E*(*Y*|*t*) from the original discrete measurement and removing the noisy observations caused by measurement errors. To define a kernel smoother, we need a bandwidth *h* and a kernel function *K*.

The Nadaraya-Watson Estimator (NW), a basic framework for kernel estimators (Nadaraya, 1964; Watson, 1964; Cai, 2001; Racine and Li, 2004; Bailey and Addison, 2010; Demir and Toktamiş, 2010; Kato, 2012; Simonoff, 2012), was defined by

(8) $$\begin{array}{l} \frac{\sum_{i=1}^{n}K_{h}\left(t-t_{i}\right)Y_{i}}{\sum_{j=1}^{n}K_{h}\left(t-t_{j}\right)}, \end{array}$$

where *K*_*h*_(*t*) = 1∕*hK*(*t*∕*h*). The kernel function *K*(*t*) was a non-negative symmetric real valued integrable function satisfying $\overset{\infty }{\underset{-\infty }{\int }}K\left(t\right)dt=1,\overset{\infty }{\underset{-\infty }{\int }}tK\left(t\right)dt=0$, and $\overset{\infty }{\underset{-\infty }{\int }}t^{2}K\left(t\right)dt>0$. The Epanechnikov kernel *K*(*t*) = 3∕4(1−*t*^2^)*I*(|*t*| < 1) was used. The bandwidth *h* controled the number of points that neighbored each *t*_*i*_ and hence determined the weight of each point contributing to the estimator. The choice of bandwidth was crucial in changing the result because it served as a smoothing parameter and determined the trade-off between the variance and bias of the resulting nonparametric regression estimates. Typically, smaller *h* decreases the bias but increases the estimation variance. We chose the optimal bandwidth that minimized the Generalized Cross Validation (GCV).

$$GCV\left(h\right)=\frac{1}{T{\left(1-\nu ∕T\right)}^{2}}\overset{T}{\underset{k=1}{\sum }}{\left(Y_{ikc}-X_{ic}\left(t_{k}\right)\right)}^{2},$$

for each subject *i* and group *c*. Here ν is the trace of matrix *M*

$$M=\left(\begin{array}{cccc} l_{1}(t_{1}) & l_{2}(t_{2}) & \ldots & l_{T}(t_{1})\\ l_{1}(t_{2}) & l_{2}(t_{2}) & \ldots & l_{T}(t_{2})\\ \vdots & \vdots & \vdots & \vdots \\ l_{1}(t_{T}) & l_{2}(t_{T}) & \ldots & l_{T}(t_{T}) \end{array}\right),$$

with

$$l_{i}\left(t\right)=\frac{K_{h}\left(t-t_{i}\right)}{\sum_{j=1}^{n}K_{h}\left(t-t_{j}\right)}$$

Once the smooth trajectory of each *X*_*i*_, *i* = 1, …, *n* was estimated from the NW nonparametric kernel smoother with the optimal bandwidth, we estimated the mean ${\hat{\mu }}_{c}\left(t\right)$ for each group directly from the sample mean, which was a consistent and unbiased estimator.

### 2.8. Hypothesis tests

The main goal of this article was to determine whether FDA applied to fNIRS data would reveal significant differences in the hemodynamic function curves between the case and control groups as they processed syntax-related stimuli. We examined eight parasylvian brain regions: left and right *inferior frontal cortex*, the *temporal parietal junction, inferior posterior parietal cortex*, and *superolateral temporal cortex*. Statistically, we used formal hypothesis tests to judge the extent to which the distributions of the random functions *X*_1*c*_, …, *X*_*nc*_ differed for case and control groups. By way of the empirical Karhunen-Loeve decompositions Equation (4), we approximated the functions of *X*_*ic*_(*t*) as

(9) $$\begin{array}{l} X_{ic}\left(t\right)={\hat{\mu }}_{c}\left(t\right)+\overset{L}{\underset{l=1}{\sum }}{\hat{\zeta }}_{ilc}{\hat{v}}_{lc}\left(t\right),c=1,2;\;i=1,\ldots ,n. \end{array}$$

As a result, the possible differences of the hemodynamic signals between the case and control group could be tested from the following three steps.

The first test was whether or not significant differences existed for the overall mean trends between case and control group for each syntax type at each brain area of interest:

$$H_{01}:\mu_{1}\left(t\right)=\mu_{2}\left(t\right),t\in T.$$

If *H*_01_ failed to be rejected, it would mean that the overall mean trends of hemodynamic curves were similar between the case and control groups. The second test was whether or not significant differences existed for the variation trends between case and control groups for each syntax type at each brain area of interest:

$$H_{0,2l}:v_{l1}\left(t\right)=v_{l2}\left(t\right),\;t\in T;l=1,\ldots ,L.$$

If *H*_0, 2*l*_ failed to be rejected, it would mean that the *l*^*th*^ variation mode had similar trends between the case and control groups. The third test was whether or not significant differences existed for the variance of principal component scores for each syntax type at each brain area of interest:

$$H_{0,3l}:\lambda_{l1}=\lambda_{l2},\;l=1,\ldots ,L.$$

If *H*_0, 3*l*_ failed to be rejected, it would mean that distribution of the *l*^*th*^ principal component scores were similar between the case and control group.

The first two tests, *H*_01_ and *H*_0, 2*l*_ were challenging because they were based on high dimensional curves, and both the test statistics and the distribution were unknown. The most traditional approach involves judging the similarity of two curves by measuring how far the norm of the differences of the two vectors is away from zero. Define the following measures (Benko et al., 2009):

$$\begin{array}{l} D_{1}=||{\hat{\mu }}_{1}\left(t\right)-{\hat{\mu }}_{2}\left(t\right)|{|}^{2}, \end{array}$$

$$\begin{array}{l} D_{2,l}=||{\hat{v}}_{l1}\left(t\right)-{\hat{v}}_{l2}\left(t\right)|{|}^{2},l=1,\ldots ,L, \end{array}$$

$$\begin{array}{l} D_{3,l}=|{\hat{\lambda }}_{l1}-{\hat{\lambda }}_{l2}{|}^{2},l=1,\ldots ,L. \end{array}$$

The three null-hypotheses would be rejected respectively, if

$$D_{1}\geq \Delta_{1;1-\alpha };\;D_{2,l}\geq \Delta_{2,l;1-\alpha };\;D_{3,l}\geq \Delta_{3,l;1-\alpha },$$

where Δ_1;1−α_, Δ_2, *l*; 1−α_, and Δ_3, *l*; 1−α_ denotes the α-level critical values of the distributions of

$$\begin{array}{l} \Delta_{1}=||\left({\hat{\mu }}_{1}\left(t\right)-\mu_{1}\left(t\right)\right)-\left({\hat{\mu }}_{2}\left(t\right)-\mu_{2}\left(t\right)\right)|{|}^{2}, \end{array}$$

$$\begin{array}{l} \Delta_{2,l}=||\left({\hat{v}}_{l1}\left(t\right)-v_{l1}\left(t\right)\right)-\left({\hat{v}}_{l2}\left(t\right)-v_{l2}\left(t\right)\right)|{|}^{2},\;l=1,\ldots ,L, \end{array}$$

$$\begin{array}{l} \Delta_{3,l}=|\left({\hat{\lambda }}_{l1}-\lambda_{l1}\right)-\left({\hat{\lambda }}_{l2}-\lambda_{l2}\right){|}^{2},\;l=1,\ldots ,L. \end{array}$$

We decided to use Δs as the primary test because *D*s were equal to Δs under the null hypotheses and the values of *D*s were shifted by the difference in the true means, eigenfunctions, and eigenvalues under the alternative hypotheses. However, because the true population mean, eigenvalues and eigenfunctions were unknown, above Δs can not be accessed directly. Therefore, we used the bootstrap sampling to determine the threshold (Benko et al., 2009).

$$\begin{array}{l} \Delta_{1}^{*}=||\left({\hat{\mu }}_{1}\left(t\right)-{\hat{\mu }}_{1}^{*}\left(t\right)\right)-\left({\hat{\mu }}_{2}\left(t\right)-{\hat{\mu }}_{2}^{*}\left(t\right)\right)|{|}^{2}, \end{array}$$

$$\begin{array}{l} \Delta_{2,l}^{*}=||\left({\hat{v}}_{l1}\left(t\right)-{\hat{v}}_{l1}^{*}\left(t\right)\right)-\left({\hat{v}}_{l2}\left(t\right)-{\hat{v}}_{l2}^{*}\left(t\right)\right)|{|}^{2},\;l=1,\ldots ,L, \end{array}$$

$$\begin{array}{l} \Delta_{3,l}^{*}=|\left({\hat{\lambda }}_{l1}-{\hat{\lambda }}_{l1}^{*}\right)-\left({\hat{\lambda }}_{l2}-{\hat{\lambda }}_{l2}^{*}\right){|}^{2},\;l=1,\ldots ,L, \end{array}$$

where ${\hat{\mu }}_{1}^{*}\left(t\right),{\hat{v}}_{l1}^{*}\left(t\right),{\hat{\lambda }}_{l1}^{*}\left(t\right)$, as well as ${\hat{\mu }}_{2}^{*}\left(t\right),{\hat{v}}_{l2}^{*}\left(t\right),{\hat{\lambda }}_{l2}^{*}\left(t\right)$ were estimated from each independent bootstrap samples $X_{11}^{*}\left(t\right),\ldots ,X_{n1}^{*}\left(t\right)$ and $X_{12}^{*}\left(t\right),\ldots ,X_{n2}^{*}\left(t\right)$, respectively. We performed 1000 nonparametric bootstrap samples for both case and control group and we repeated the nonparametric kernel smoothing for each sample. Finally the 1−α percentiles were used to determine the thresholds of the tests.

## 3. Results

### 3.1. Real NIRS data analysis

Behaviorally, the case (SLI) group identified the agents of subject-verb-object and subject relative clause sentences as well as their age-matched, typically developing controls. However, the children in the case group were significantly less accurate than the children in the control group on the passive and object relative clause sentences.

The goal of statistical modeling was to determine whether there were significant differences in the hemodynamic trends between the case and control groups. Additionally, we speculated which brain regions were associated with children's syntax comprehension ability from the significant group differences. For each hemodynamic category (Δ*HbO* and Δ*HbD*), we performed 32 tests to consider all combinations of four different syntax types and eight different brain regions.

Using the FDA approaches described in Sections 2.5 and 2.6, we first estimated the mean function ${\hat{\mu }}_{c}\left(t\right)$, eigenfunctions ${\hat{v}}_{lc}\left(t\right)$, and eigenvalues ${\hat{\lambda }}_{lc}$ for each group, with *c* = 1 corresponding to case group and *c* = 2 for control group. During the analysis, we kept the first two eigenfunctions (i.e., *L* = 2) because they explained 90% of the overall variations, and the remaining eigenfunctions explained only a very small percentage of the variations.

**Figure 5:**
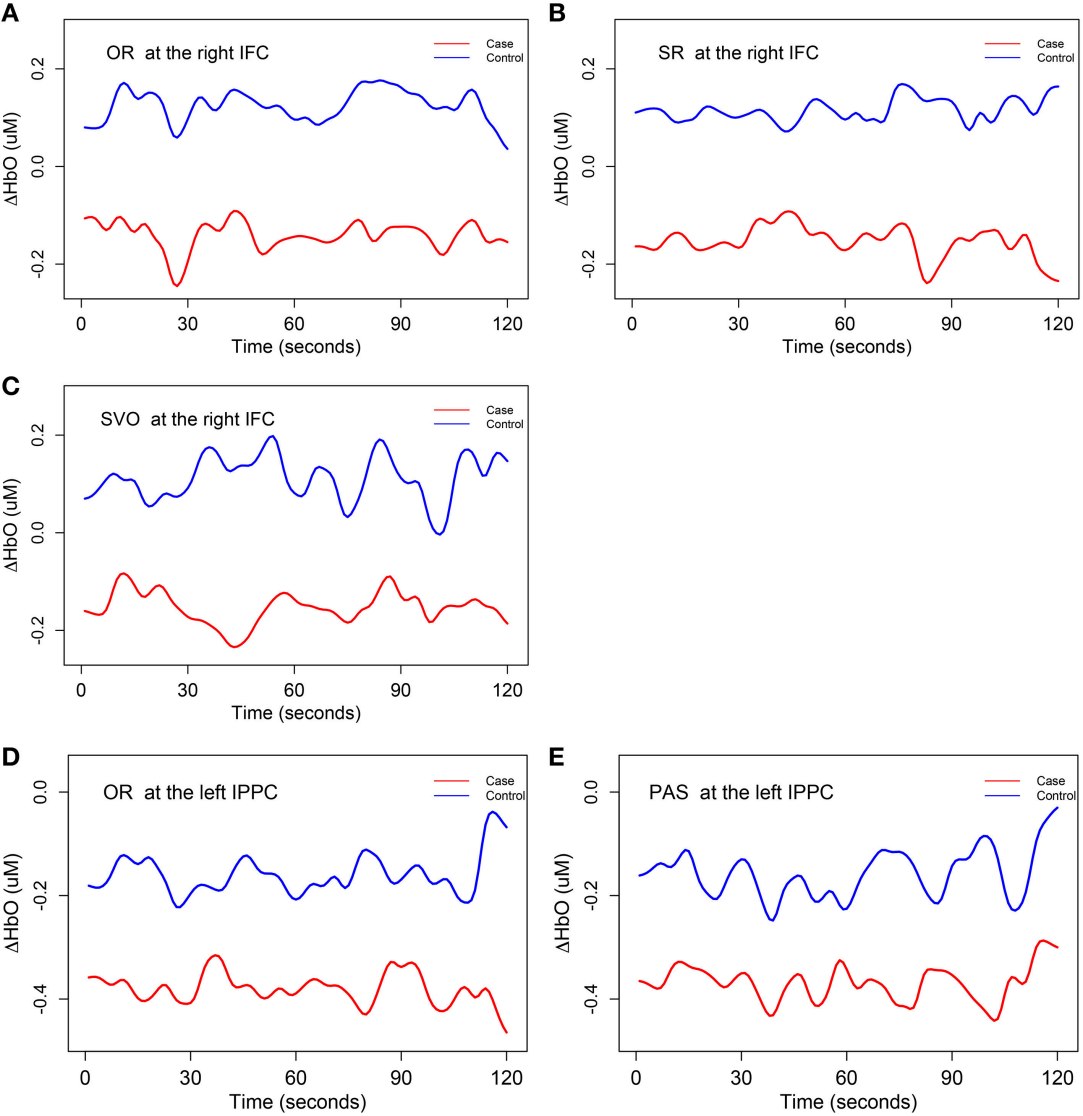
**The mean trends for Δ***HbO*** (i.e., ***H***_**01**_)**. The mean trajectories ${\hat{\mu }}_{c}\left(t\right)$ of 15 smoothing Δ*HbO* curves, in the control group are depicted as a blue line and similar information for case group are depicted as a red line. IFC, inferior frontal cortex; IPPC, inferior posterior parietal cortex. OR, object relative clause sentences; SR, subject relative clause sentences; SVO, subject-verb-object sentences; and PAS, passive sentences. **(A)** The mean trajectories of Δ*HbO* for OR syntax type at the right IFC; **(B)** the mean trajectories of Δ*HbO* for SR syntax type at the right IFC; **(C)** the mean trajectories of Δ*HbO* for SVO syntax type at the right IFC; **(D)** the mean trajectories of Δ*HbO* for OR syntax type at the left IPPC; **(E)** the mean trajectories of Δ*HbO* for PAS syntax type at the left IPPC.

With respect to potential group differences in mean trends of Δ*HbO*, *H*_01_ was rejected at the significance level of 0.1 at two brain regions: right *inferior frontal cortex* brain region for subject-verb-object, subject relative clause, and object relative clause sentences, and at the left *inferior posterior parietal cortex* brain region for object relative clause and passive sentences. Therefore, we concluded that the right *inferior frontal cortex* and left *inferior posterior parietal cortex* were associated with the control group's syntax comprehension processing ability to a greater extent than the SLI group's. Figure 5 displays the estimated mean trajectories ${\hat{\mu }}_{c}\left(t\right)$ of Δ*HbO* in these two brain regions with corresponding significant syntax types. A close inspection of Figure 5 reveals that the mean trajectories of case and control groups have different dynamic trends (different shape and magnitude) for each syntax type, with opposite fluctuate oscillations at some time segments but similar directions at other time segments. The mean trajectories of the control group were always above those of the case group in these two brain regions. In the right *inferior frontal cortex* brain region, the mean oxygenated hemodynamic trajectories of the control group were always above zero, while those of the case group were below zero. In the left *inferior posterior partietal cortex*, the mean oxygenated hemodynamic trajectories of both case and control groups were below zero.

**Figure 6:**
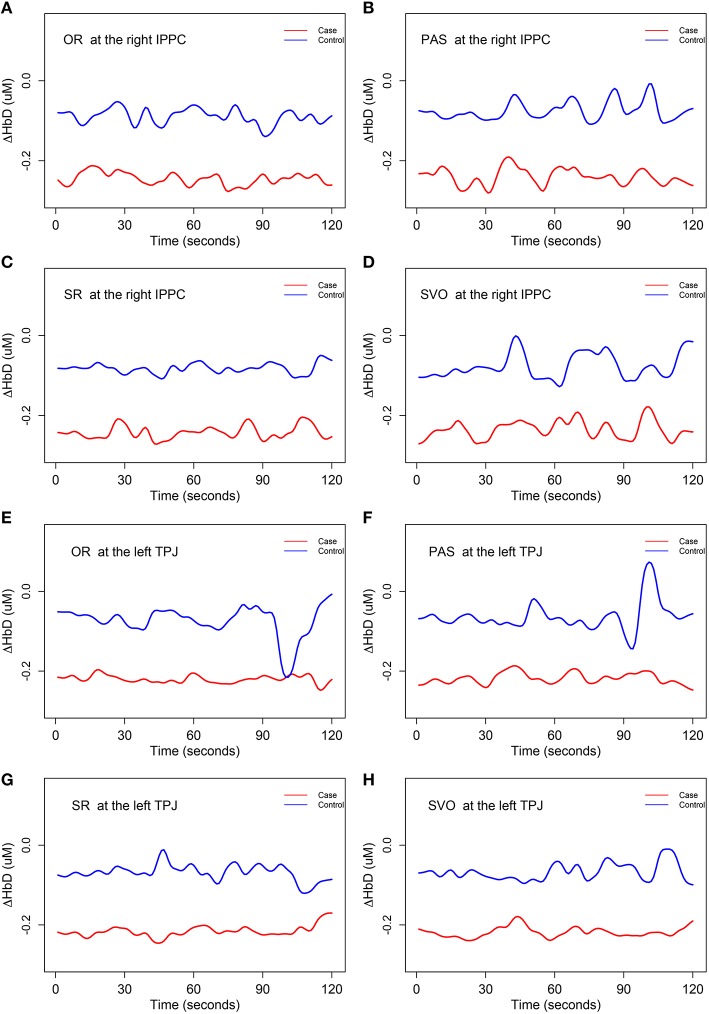
**The mean trends for Δ***HbD*** (i.e., ***H***_**01**_)**. IPPC, inferior posterior parietal cortex; TPJ, temporal parietal junction. OR, object relative clause sentences; SR, subject relative clause sentences; SVO, subject-verb-object sentences; and PAS, passive sentences. **(A)** The mean trajectories of Δ*HbD* for OR syntax type at the right IPPC; **(B)** the mean trajectories of Δ*HbD* for PAS syntax type at the right IPPC; **(C)** the mean trajectories of Δ*HbD* for SR syntax type at the right IPPC; **(D)** the mean trajectories of Δ*HbD* for SVO syntax type at the right IPPC; **(E)** the mean trajectories of Δ*HbD* for OR syntax type at the left TPJ; **(F)** the mean trajectories of Δ*HbD* for PAS syntax type at the left TPJ; **(G)** the mean trajectories of Δ*HbD* for SR syntax type at the left TPJ; **(H)** the mean trajectories of Δ*HbD* for SVO syntax type at the left TPJ.

The hypothesis test *H*_01_ for Δ*HbD* was rejected at the right *inferior posterior parietal cortex* (at 0.05 significance level) and the left *temporal parietal junction* (at 0.1 significance level) for all four syntax types. We concluded that there were significant differences (related to both shape and magnitude) in the mean trajectories of Δ*HbD* at these two brain regions between case and control group, and these two brain regions were also associated with children's syntax comprehension ability. A close inspection of Figure 6 reveals that the mean trajectories of Δ*HbD* for the control group mainly fluctuate around zero but those of the case group are around −0.2 for all the eight scenarios. Using the same range of y-axis as the oxygenated hemodynamic trajectories of Δ*HbO* in Figure 5, the overall mean trends of the deoxygenated hemodynamic trajectories Δ*HbD* were very flat, especially those of the case group. So, we decreased the range of the y-axis in Figure 6 to the half of that of Figure 5 so that the significant oscillations were more apparent.

None of the 32 hypothesis tests related to the variation trends (*H*_0, 2*l*_, *l* = 1, 2) could be rejected for either Δ*HbO* or Δ*HbD* at any of the eight brain regions or for any of the four syntax type types. Thus, there were no significant differences in the eigenfunction (i.e., variation trends) in Δ*HbD* and Δ*HbD* between the case and control groups.

Hypothesis test *H*_0, 3*l*_, *l* = 1, 2, related to the eigenvalues, was rejected at a few brain regions and syntax types. It indicated that the percentages of variation explained by the first two eigenfunctions (i.e., the distributions of the first two principle component scores) were significantly different between case and control groups. Table 1 summarizes the details of percentage of variation for all significant brain regions and syntax types. Among all these significant differences, the left *inferior posterior parietal cortex* brain region for Δ*HbD* achieved the maximum for all four syntax types, with the first eigenfunction of the case group (*v*_11_(*t*)) explaining 96−98% of the total variation of the case group vs. 59−70% of the total variation of the control group (*v*_12_(*t*)). Similarly, the second eigenfunction (*v*_21_(*t*)) explained 0.7−1.0% of the total variation of the case group vs. 15−34% of the total variation of the control group (*v*_22_(*t*)). Additionally, we also noticed that the *superolateral temporal cortex* brain regions for Δ*HbO* showed opposite directions in the percentage of variation explained by the first two eigenfuncitons as compared to other brain regions. Specifically, the first eigenfunction of the case group (*v*_11_(*t*)) explained a greater percentage of total variation than the first eigenfunction of the control group (*v*_12_(*t*)) for almost all scenarios, except the Δ*HbO* at left *superolateral temporal cortex* for passive and subject-verb-objects sentences, and right *superolateral temporal cortex* for object relative clause sentences. Also, we observed that the second eigenfunction of the case group (*v*_21_(*t*)) explained a much smaller percentage of total variation than the second eigenfunction of the control group (*v*_22_(*t*)) for almost all scenarios with the exception of the Δ*HbO* at left *superolateral temporal cortex* for subject-verb-object sentences and right *superolateral temporal cortex* for object relative clause sentences.

**Table 1:** **Significant group differences in the percentages of variation explained by the first two eigenfunctions between the case and control groups**.

**Category**	**Brain region**	**Syntax**	**Case**		**Control**	
			***v*_11_(%)**	***v*_21_(%)**	***v*_12_(%)**	***v*_22_(%)**
Δ*HbO*	left IPPC	OR	88.3	5.5	76.8	15.3
Δ*HbO*	left TPJ	PAS	97.4	0.6	87.9	5.2
Δ*HbO*	left STC	PAS	89.8	3.9	92.8	5.4
Δ*HbO*	left STC	SVO	84.7	6.0	95.2	1.8
Δ*HbO*	right STC	OR	83.0	6.2	96.1	1.3
Δ*HbD*	left IPPC	OR	97.5	0.9	59.8	33.6
Δ*HbD*	left IPPC	PAS	97.6	0.8	61.2	31.4
Δ*HbD*	left IPPC	SR	97.7	0.7	66.8	23.8
Δ*HbD*	left IPPC	SVO	96.8	1.0	69.8	15.6
Δ*HbD*	left TPJ	OR	97.1	0.8	83.0	12.8
Δ*HbD*	left TPJ	PAS	97.1	0.8	87.6	8.3
Δ*HbD*	left TPJ	SR	97.9	0.6	91.8	4.5
Δ*HbD*	left STC	OR	93.8	2.4	83.5	13.3
Δ*HbD*	left STC	PAS	92.1	2.7	87.7	10.0
Δ*HbD*	left STC	SR	95.0	1.9	88.8	8.1
Δ*HbD*	left STC	SVO	92.4	2.6	89.2	5.5

*IPPC, inferior posterior parietal cortex; TPJ, temporal parietal junction; STC, superolateral temporal cortex; OR, object relative clause sentences; SR, subject relative clause sentences; SVO, subject-verb-object sentences; and PAS, passive sentences; v_11_ stands for the first eigenfunction of the case group; v_21_ stands for the second eigenfunction of the case group; v_12_ stands for the first eigenfunction of the control group; and v_22_ stands for the second eigenfunction of the control group*.

## 4. Discussion

The primary goal of this article was to determine whether significant group differences in the hemodynamic trajectories existed for two groups with known language differences. To achieve this goal, we designed a syntax comprehension task in which 15 children with SLI and 15 age-matched, typically-developing controls pointed to pictures representing the agent (actor) after hearing four types of sentences (subject-verb-object sentences, subject relative clause sentences, passive sentences, and object relative clause sentences). We administered the 60 questions in a pseudo-random order to 30 participants during the NIRS data collection. We performed three formal hypothesis tests to formally assess the group differences between the case and control groups, and determined the threshold by a bootstrap approach for high dimensional objects when both test statistics and distributions were unknown (Benko et al., 2009).

The FDA approach is different from the widly used traditional approaches in existing NIRS literature (e.g., GLM and *t*-test). In functional data analyis, the modeling is performed in the functional sense that treats the entire curve as the modeling target and fully utilizes the superior temporal resolution of fNIRS data. But GLM extracts multivariate discrete points and does not utilize the dynamic trajectories of the fNIRS curve. As a nonparametric data-driven approach, FDA does not assume any linear structure or normality distribution such as that within the GLM model (Shimada and Hiraki, 2006; Koh et al., 2007; Abdelnour and Huppert, 2009; Custo et al., 2010; Penny et al., 2011; Tak and Ye, 2014). Unlike simple *t*-tests (Aldrich et al., 1994; Germon et al., 1994, 1999; Young et al., 2000; Hoshi et al., 2001; Isobe et al., 2001; Kennan et al., 2002; Schroeter et al., 2002; Hoshi, 2003; Matsuo et al., 2003; Tachtsidis et al., 2004; Tsujimoto et al., 2004; Shibuya-Tayoshi et al., 2007; Kim et al., 2010), FDA tests the trajectory differences of two entire curves for two groups and captures not only the differences in magnitude but also in shape. Thus, our approach was inclusive of all observed stimulus-relevant data information and was not restricted to the magnitudee differences as *t*-test does.

We successfully detected significant group differences in the oxygenated hemodynamic mean trends in two brain regions, right *inferior frontal cortex* and left *inferior posterior parietal cortex*. The mean oxygenated hemodynamic trajectories between case and control groups showed different trends (different shape and magnitude) in these two brain regions, with some segments showing opposite fluctuating oscillations but other segments having similar directions. In the right *inferior frontal cortex*, the mean oxygenated hemodynamic trajectories of the control group were always above zero, while those of the case group were below zero. In the left *inferior posterior partietal cortex*, the mean oxygenated hemodynamic trajectories of both case and control groups were below zero. We also detected significant group differences in deoxygenated hemodynamic mean trends in the right *inferior posterior partietal cortex* and left *temporal parietal junction*. The mean deoxygenated hemodynamic trajectories of the control group mainly fluctuated around the zero line while that of case group were all below −0.2. Some of these significant findings from our quantitative functional NIRS analysis were consistent with the results of a few other studies that had dramatically different approaches, experiments, data sets, and foci. For example, the left *inferior posterior parietal cortex (Angular Gyrus)* brain region has been reported to be highly engaged in semantic processing during language comprehension (Geschwind, 1965; Joseph, 1982; Démonet et al., 1992; Vandenberghe et al., 1996; Vigneau et al., 2006; Houdé et al., 2010; Price, 2010), including some reports got by MRI (Binder et al., 2009; Seghier et al., 2010; Seghier, 2013). Further, differences between children with and without SLI in the extent of activation of this area has been noted in studies of listening to nonwords and words (Weismer et al., 2005). A number of MRI studies have noted group differences between children with SLI and their age-matched controls in the size of right hemisphere parasylvian areas (Plante et al., 1991).

There were no significant differences in the eigenfunctions, but the percentage of total variation explained by each eigenfunction significantly differenced in the left *inferior posterior partietal cortex*, left *temporal parietal junction*, and both left and right *superior temporal cortex*. The finding of significant group differences in the percentage of variation explained by the first two eigenfunctions may be of particular interest. Recall that the first two orthogonal eigenfunctions derived from the fNIRS high dimensional auto covariance matrix were likely related to the cognitive processes involved in performing our syntax comprehension task. The significant group differences in the percentage of total variation explained by the eigenfunctions may relate to group differences in information processing functions that have been associated with attention, semantic processing, and syntactic processing in the left *inferior posterior parietal cortex*, the left *temporal parietal junction*, and the left *superolateral temporal cortex*. Further research on larger samples of participants are needed to fully understand the meaning of these results.

In future work, we will compare the significant differences between left and right hemispheres. Unlike the comparisons between case and control groups, the left and right brain samples are not independent requiring a different approach. We will also explore more detailed functional properties in the rest periods. Although there are several hypothesis tests involved, we will leave the multiple correction for the future for a few reasons. First, there are only 15 subjects within each group, which is much less than the dimension of the curves (length of 120 after preprocessing and length of 8521 before preprocessing). As a result, power is limited due to the difficulties of collecting children with SLI. Therefore, we do not want to diminish our findings due to a large number of multiple corrections. We believe that our methods will yield better results after the sample size is large enough and will investigate the multiple correction when we have an appropriate sample size. Second, the multiple tests involved here are not independent. Instead, they form close correlations, as Δ*HbO* and Δ*HbD* and the four syntax types are highly correlated. Therefore, many multiple correction approaches will not be appropriate and likely will mislead the results. For example, the test of equal mean hemodynamic trends between case and control (*H*_1, 0_) was rejected, whether we considered each of the syntax types (with 120 length) individually or we tested the stimuli of the four syntax types simultaneously (with 480 length). However, if we used multiple correction, say Bonferroni correction, then each syntax test would only have an α∕4 significance level, which makes the individual syntax period impossible to be rejected given the current sample size. In that case, none of the individual syntax types would show significant differences, but the whole stimuli curve with four syntax types would be significant between case and control. This would result in conflicting conclusions.

In summary, this proof of concept study was conducted to explore a more advanced statistical analysis approach to the analysis of the time course of hemodynamic data collected with fNIRS. This approach enables us to compare which brain regions are significantly involved in syntax comprehension ability in the two groups. FDA strategies were used to decompose the high dimensional Δ*HbO* and Δ*HbD* time curves into mean curves and eigenfunctions to represent overall trends and variation structures (Ramsay and Silverman, 2002; Ferraty and Vieu, 2006; Ramsay, 2006; Barati et al., 2013). After detailed comparisons and hypothesis tests, we revealed greater brain activity for the control group than the case group for all four syntax types. In addition, different percentages of variation for the case and control groups were explained by the first two eigenfunctions, suggesting that the two groups used different cognitive processing strategies while performing the tasks. The approach of FDA proposed in this paper has promise as an analysis method that captures the overall mean trends and variation trends of hemoglobin concentration over time within and between groups without assuming any structure.

## Ethics statement

This study was approved by the Utah State University Institutional Review Board. All participants (adults and children) and the parents or guardians of all children signed consent forms that were approved by the IRB. The participant's name “Sue” was a pseudonym.

## Author contributions

GF conceived the statistical modeling, programmed and performed the data analysis and figures, and wrote the first version of the manuscript; NW collected and processed the data; JB assisted with experimental design and programmed the task; JM and JE created the experimental task; RG conceived the research, helped design the experimental task, supervised all aspects of data collection and data processing, edited the manuscript, and wrote the experimental design and discussion sections.

## Funding

Funded in part by the Lillywhite Endowment to Utah State University.

### Conflict of interest statement

The authors declare that the research was conducted in the absence of any commercial or financial relationships that could be construed as a potential conflict of interest.

## References

[B1] Abdelnour A. F. Huppert T. (2009). Real-time imaging of human brain function by near-infrared spectroscopy using an adaptive general linear model. Neuroimage 46, 133–143. 10.1016/j.neuroimage.2009.01.033 19457389 PMC2758631

[B2] Akgül C. B. Akin A. Sankur B. (2006). Extraction of cognitive activity-related waveforms from functional near-infrared spectroscopy signals. Med. Biol. Eng. Comput. 44, 945–958. 10.1007/s11517-006-0116-3 17061116

[B3] Aldrich C. J. Wyatt J. S. Spencer J. A. Reynolds E. O. Delpy D. T. (1994). The effect of maternal oxygen administration on human fetal cerebral oxygenation measured during labour by near infrared spectroscopy. Br. J. Obstet. Gynaecol. 101, 509–513. 8018640 10.1111/j.1471-0528.1994.tb13152.x

[B4] Arenth P. M. Ricker J. H. Schultheis M. T. (2007). Applications of functional near-infrared spectroscopy (fNIRS) to neurorehabilitation of cognitive disabilities. Clin. Neuropsychol. 21, 38–57. 10.1080/13854040600878785 17366277

[B5] Bailey R. W. Addison J. T. (2010). A smoothed-distribution form of nadaraya-watson estimation, Department of Economics Discussion Paper (Coimbra), 10–30.

[B6] Barati Z. Zakeri I. Pourrezaei K. (2013). Functional data analysis view of functional near infrared spectroscopy data. J. Biomed. Opt. 18, 117007. 10.1117/1.JBO.18.11.117007 24247748

[B7] Bartocci M. Winberg J. Ruggiero C. Bergqvist L. L. Serra G. Lagercrantz H. (2000). Activation of olfactory cortex in newborn infants after odor stimulation: a functional near-infrared spectroscopy study. Pediatr. Res. 48, 18–23. 10.1203/00006450-200007000-00006 10879795

[B8] Ben Schachar M. Hendler T. Kahn I. Ben Bashat D. Grodzinsky Y. (2003). The neural reality of syntactic transformations. Psychol. Sci. 14, 433–440. 10.1111/1467-9280.01459 12930473

[B9] Ben-Shachar M. Palti D. Grodzinsky Y. (2004). Neural correlates of syntactic movement: converging evidence from two fMRI experiments. Neuroimage 21, 1320–1336. 10.1016/j.neuroimage.2003.11.027 15050558

[B10] Benko M. Härdle W. Kneip A. (2009). Common functional principal components. Annal. Stat. 37, 1–34. 10.1214/07-AOS516

[B11] Binder J. R. Desai R. H. Graves W. W. Conant L. L. (2009). Where is the semantic system? A critical review and meta-analysis of 120 functional neuroimaging studies. Cereb. Cortex 19, 2767–2796. 10.1093/cercor/bhp055 19329570 PMC2774390

[B12] Bishop D. (2014). Ten questions about terminology for children with unexplained language problems. Int. J. Lang. Commun. Disord. 49, 381–415. 10.1111/1460-6984.12101 25142090 PMC4314704

[B13] Boas D. A. Elwell C. E. Ferrari M. Taga G. (2014). Twenty years of functional near-infrared spectroscopy: introduction for the special issue. Neuroimage 85, 1–5. 10.1016/j.neuroimage.2013.11.033 24321364

[B14] Cai Z. (2001). Weighted nadaraya–watson regression estimation. Stat. Probab. Lett. 51, 307–318. 10.1016/S0167-7152(00)00172-3

[B15] Cui X. Bray S. Reiss A. L. (2010). Functional near infrared spectroscopy (NIRS) signal improvement based on negative correlation between oxygenated and deoxygenated hemoglobin dynamics. Neuroimage 49, 3039–3046. 10.1016/j.neuroimage.2009.11.050 19945536 PMC2818571

[B16] Custo A. Boas D. A. Tsuzuki D. Dan I. Mesquita R. Fischl B.. (2010). Anatomical atlas-guided diffuse optical tomography of brain activation. Neuroimage 49, 561–567. 10.1016/j.neuroimage.2009.07.033 19643185 PMC2858333

[B17] Dehghani H. Delpy D. T. (2000). Near-infrared spectroscopy of the adult head: effect of scattering and absorbing obstructions in the cerebrospinal fluid layer on light distribution in the tissue. Appl. Opt. 39, 4721–4729. 10.1364/AO.39.004721 18350064

[B18] Demir S. Toktamiş Ö. (2010). On the adaptive nadaraya-watson kernel regression estimators. Hacettepe J. Math. Stat. 39, 429–437.

[B19] Démonet J.-F. Chollet F. Ramsay S. Cardebat D. Nespoulous J.-L. Wise R.. (1992). The anatomy of phonological and semantic processing in normal subjects. Brain 115, 1753–1768. 10.1093/brain/115.6.1753 1486459

[B20] Dick F. Wulfeck B. Krupa-Kwiatkowski M. Bates E. (2004). The development of complex sentence interpretation in typically developing children compared with children with specific language impairments or early unilateral focal lesions. Dev. Sci. 7, 360–377. 10.1111/j.1467-7687.2004.00353.x 15595375

[B21] Fallgatter A. J. Strik W. K. (1998). Frontal brain activation during the wisconsin card sorting test assessed with two-channel near-infrared spectroscopy. Eur. Arch. Psychiatry Clin. Neurosci. 248, 245–249. 10.1007/s004060050045 9840371

[B22] Fallgatter A. J. Strik W. K. (2000). Reduced frontal functional asymmetry in schizophrenia during a cued continuous performance test assessed with near-infrared spectroscopy. Schizophr. Bull. 26, 913–919. 10.1093/oxfordjournals.schbul.a033505 11087023

[B23] Ferraty F. Vieu P. (2006). Nonparametric Functional Data Analysis: Theory and Practice. New York, NY: Springer Science & Business Media.

[B24] Folley B. S. Park S. (2005). Verbal creativity and schizotypal personality in relation to prefrontal hemispheric laterality: a behavioral and near-infrared optical imaging study. Schizophr. Res. 80, 271–282. 10.1016/j.schres.2005.06.016 16125369 PMC2817946

[B25] Fujiwara N. Sakatani K. Katayama Y. Murata Y. Hoshino T. Fukaya C.. (2004). Evoked-cerebral blood oxygenation changes in false-negative activations in BOLD contrast functional MRI of patients with brain tumors. Neuroimage 21, 1464–1471. 10.1016/j.neuroimage.2003.10.042 15050571

[B26] Germon T. J. Evans P. D. Barnett N. J. Wall P. Manara A. R. Nelson R. J. (1999). Cerebral near infrared spectroscopy: emitter-detector separation must be increased. Br. J. Anaesth. 82, 831–837. 10562774 10.1093/bja/82.6.831

[B27] Germon T. J. Kane N. M, Manara, A. R. Nelson R. J. (1994). Near-infrared spectroscopy in adults: effects of extracranial ischaemia and intracranial hypoxia on estimation of cerebral oxygenation. Br. J. Anaesth. 73, 503–506. 7999492 10.1093/bja/73.4.503

[B28] Geschwind N. (1965). Disconnexion syndromes in animals and man. Brain 88, 585. 5318824 10.1093/brain/88.3.585

[B29] Grodzinsky Y. (2000). The neurology of syntax: language use without broca's area. Behav. Brain Sci. 23, 1–21. 10.1017/S0140525X00002399 11303337

[B30] Hall M. Chaudhary U. Rey G. Godavarty A. (2013). Fronto-temporal mapping and connectivity using NIRS for language-related paradigms. J. Neurolinguistics 26, 178–194. 10.1016/j.jneuroling.2012.06.002

[B31] Herrmann M. J. Ehlis A.-C. Fallgatter A. J. (2003). Frontal activation during a verbal-fluency task as measured by near-infrared spectroscopy. Brain Res. Bull. 61, 51–56. 10.1016/j.jneuroling.2012.06.002 12788206

[B32] Hoge R. D. Franceschini M. A. Covolan R. J. Huppert T. Mandeville J. B. Boas D. A. (2005). Simultaneous recording of task-induced changes in blood oxygenation, volume, and flow using diffuse optical imaging and arterial spin-labeling MRI. Neuroimage 25, 701–707. 10.1016/j.neuroimage.2004.12.032 15808971

[B33] Hoshi Y. (2003). Functional near-infrared optical imaging: utility and limitations in human brain mapping. Psychophysiology 40, 511–520. 10.1111/1469-8986.00053 14570159

[B34] Hoshi Y. Kobayashi N. Tamura M. (2001). Interpretation of near-infrared spectroscopy signals: a study with a newly developed perfused rat brain model. J. Appl. Physiol. 90, 1657–1662. 11299252 10.1152/jappl.2001.90.5.1657

[B35] Houdé O. Rossi S. Lubin A. Joliot M. (2010). Mapping numerical processing, reading, and executive functions in the developing brain: an fMRI meta-analysis of 52 studies including 842 children. Dev. Sci. 13, 876–885. 10.1111/j.1467-7687.2009.00938.x 20977558

[B36] Huppert T. J. Diamond S. G. Franceschini M. A. Boas D. A. (2009). Homer: a review of time-series analysis methods for near-infrared spectroscopy of the brain. Appl. Opt. 48, 280–298. 10.1364/AO.48.00D280 19340120 PMC2761652

[B37] Irani F. Platek S. M. Bunce S. Ruocco A. C. Chute D. (2007). Functional near infrared spectroscopy (fNIRS): an emerging neuroimaging technology with important applications for the study of brain disorders. Clin. Neuropsychol. 21, 9–37. 10.1080/13854040600910018 17366276

[B38] Isobe K. Kusaka T. Nagano K. Okubo K. Yasuda S. Kondo M.. (2001). Functional imaging of the brain in sedated newborn infants using near infrared topography during passive knee movement. Neurosci. Lett. 299, 221–224. 10.1016/S0304-3940(01)01518-X 11165775

[B39] Joseph R. (1982). The neuropsychology of development: hemispheric laterality, limbic language, and the origin of thought. J. Clin. Psychol. 38, 4–33. 7056873 10.1002/1097-4679(198201)38:1<4::aid-jclp2270380102>3.0.co;2-j

[B40] Kameyama M. Fukuda M. Yamagishi Y. Sato T. Uehara T. Ito M.. (2006). Frontal lobe function in bipolar disorder: a multichannel near-infrared spectroscopy study. Neuroimage 29, 172–184. 10.1016/j.neuroimage.2005.07.025 16125979

[B41] Karhunen K. (1946). Zur spektraltheorie stochastischer prozesse. Ann. Acad. Sci. Finnicae Ser. A 34, 1–7. 15812976

[B42] Kato K. (2012). Weighted nadaraya–watson estimation of conditional expected shortfall. J. Financ. Econom. 10, 265–291. 10.1093/jjfinec/nbs002

[B43] Kennan R. P. Kim D. Maki A. Koizumi H. Constable R. T. (2002). Non-invasive assessment of language lateralization by transcranial near infrared optical topography and functional MRI. Human Brain Mapp. 16, 183–189. 10.1002/hbm.10039 12112772 PMC6871823

[B44] Kim M. N. Durduran T. Frangos S. Edlow B. L. Buckley E. M. Moss H. E.. (2010). Noninvasive measurement of cerebral blood flow and blood oxygenation using near-infrared and diffuse correlation spectroscopies in critically brain-injured adults. Neurocrit. Care 12, 173–180. 10.1007/s12028-009-9305-x 19908166 PMC2844468

[B45] Koh P. H. Glaser D. E. Flandin G. Kiebel S. Butterworth B. Maki A.. (2007). Functional optical signal analysis: a software tool for near-infrared spectroscopy data processing incorporating statistical parametric mapping. J. Biomed. Opt. 12, 064010. 10.1117/1.2804092 18163826

[B46] Kozel F. A. Tian F. Dhamne S. Croarkin P. E. McClintock S. M. Elliott A.. (2009). Using simultaneous repetitive transcranial magnetic stimulation/functional near infrared spectroscopy (rTMS/fNIRS) to measure brain activation and connectivity. Neuroimage 47, 1177–1184. 10.1016/j.neuroimage.2009.05.016 19446635 PMC2728000

[B47] Leonard L. B. (2014). Children with Specific Language Impairment, 2nd Edn. Cambridge: MIT Press.

[B48] Mandeville J. B. Marota J. J. Ayata C. Moskowitz M. A. Weisskoff R. M. Rosen B. R. (1999). MRI measurement of the temporal evolution of relative CMRO 2 during rat forepaw stimulation. Magn. Reson. Med. 42, 944–951. 10542354 10.1002/(sici)1522-2594(199911)42:5<944::aid-mrm15>3.0.co;2-w

[B49] Matsuo K. Taneichi K. Matsumoto A. Ohtani T. Yamasue H. Sakano Y.. (2003). Hypoactivation of the prefrontal cortex during verbal fluency test in PTSD: a near-infrared spectroscopy study. Psychiatry Res. 124, 1–10. 10.1016/S0925-4927(03)00093-3 14511791

[B50] Montgomery J. W. Evans J. L. Gillam R. B. Sergeev A. V. Finney M. C. (2015). “Whatdunit?” Developmental changes in children's syntactically-based sentence interpretation abilities and sensitivity to word order. Appl. Psycholinguist. 1, 1–12. 10.1017/S0142716415000570

[B51] Müller H.-G. (2008). Functional modeling of longitudinal data. Longitudinal Data Anal. 1, 223–252. 10.1201/9781420011579.ch10

[B52] Müller R.-A. Kleinhans N. Courchesne E. (2003). Linguistic theory and neuroimaging evidence: an fMRI study of brocas area in lexical semantics. Neuropsychologia 41, 1199–1207. 10.1016/S0028-3932(03)00045-9 12753959

[B53] Nadaraya E. A. (1964). On estimating regression. Theory Probab. Appl. 9, 141–142. 24992657

[B54] Okamoto M. Dan H. Shimizu K. Takeo K. Amita T. Oda I.. (2004). Multimodal assessment of cortical activation during apple peeling by NIRS and fMRI. Neuroimage 21, 1275–1288. 10.1016/j.neuroimage.2003.12.003 15050555

[B55] Penny W. D. Friston K. J. Ashburner J. T. Kiebel S. J. Nichols T. E. (2011). Statistical Parametric Mapping: The Analysis of Functional Brain Images: The Analysis of Functional Brain Images. Cambridge, MA: Academic Press.

[B56] Petrides M. (2013). Neuroanatomy of Language Regions of the Human Brain. Cambridge, MA: Academic Press.

[B57] Plante E. Swisher L. Vance R. Rapcsak S. (1991). MRI findings in boys with specific language impairment. Brain Lang. 41, 52–66. 1884191 10.1016/0093-934x(91)90110-m

[B58] Plichta M. M. Heinzel S. Ehlis A.-C. Pauli P. Fallgatter A. J. (2007). Model-based analysis of rapid event-related functional near-infrared spectroscopy (NIRS) data: a parametric validation study. Neuroimage 35, 625–634. 10.1016/j.neuroimage.2006.11.028 17258472

[B59] Price C. J. (2010). The anatomy of language: a review of 100 fMRI studies published in 2009. Ann. N.Y. Acad. Sci. 1191, 62–88. 10.1111/j.1749-6632.2010.05444.x 20392276

[B60] Racine J. Li Q. (2004). Nonparametric estimation of regression functions with both categorical and continuous data. J. Econom. 119, 99–130. 10.1016/S0304-4076(03)00157-X

[B61] Ramsay J. O. (2006). Functional Data Analysis. Hoboken, NJ: Wiley Online Library.

[B62] Ramsay J. O. Silverman B. W. (2002). Applied Functional Data Analysis: Methods and Case Studies, Vol.77 New York, NY: Springer.

[B63] Rossi S. Telkemeyer S. Wartenburger I. Obrig H. (2012). Shedding light on words and sentences: near-infrared spectroscopy in language research. Brain Lang. 121, 152–163. 10.1016/j.bandl.2011.03.008 21546074

[B64] Scherer L. C. Fonseca R. P. Amiri M. Adrover-Roig D. Marcotte K. Giroux F.. (2012). Syntactic processing in bilinguals: an fNIRS study. Brain Lang. 121, 144–151. 10.1016/j.bandl.2011.09.009 22177410

[B65] Schroeter M. L. Bücheler M. M. Müller K. Uludağ K. Obrig H. Lohmann G.. (2004). Towards a standard analysis for functional near-infrared imaging. Neuroimage 21, 283–290. 10.1016/j.neuroimage.2003.09.054 14741666

[B66] Schroeter M. L. Zysset S. Kupka T. Kruggel F. Von Cramon D. Y. (2002). Near-infrared spectroscopy can detect brain activity during a color–word matching stroop task in an event-related design. Human Brain Mapp. 17, 61–71. 10.1002/hbm.10052 12203689 PMC6872032

[B67] Seghier M. L. (2013). The angular gyrus multiple functions and multiple subdivisions. Neuroscientist 19, 43–61. 10.1177/1073858412440596 22547530 PMC4107834

[B68] Seghier M. L. Fagan E. Price C. J. (2010). Functional subdivisions in the left angular gyrus where the semantic system meets and diverges from the default network. J. Neurosci. 30, 16809–16817. 10.1523/JNEUROSCI.3377-10.2010 21159952 PMC3105816

[B69] Shibuya-Tayoshi S. Sumitani S. Kikuchi K. Tanaka T. Tayoshi S. Ueno S.-I.. (2007). Activation of the prefrontal cortex during the trail-making test detected with multichannel near-infrared spectroscopy. Psychiatry Clin. Neurosci. 61, 616–621. 10.1111/j.1440-1819.2007.01727.x 18081621

[B70] Shimada S. Hiraki K. (2006). Infant's brain responses to live and televised action. Neuroimage 32, 930–939. 10.1016/j.neuroimage.2006.03.044 16679032

[B71] Siegel A. M. Culver J. P. Mandeville J. B. Boas D. A. (2003). Temporal comparison of functional brain imaging with diffuse optical tomography and fMRI during rat forepaw stimulation. Phys. Med. Biol. 48, 1391. 10.1088/0031-9155/48/10/311 12812454

[B72] Simonoff J. S. (2012). Smoothing Methods in Statistics. New York, NY: Springer Science & Business Media.

[B73] Steinbrink J. Villringer A. Kempf F. Haux D. Boden S. Obrig H. (2006). Illuminating the BOLD signal: combined fMRI–fNIRS studies. Magn. Reson. Imaging 24, 495–505. 10.1016/j.mri.2005.12.034 16677956

[B74] Strangman G. E. Zhang Q. Zeffiro T. (2009). Near-infrared neuroimaging with NinPy. Front. Neuroinform. 3:12. 10.3389/neuro.11.012.2009 19543449 PMC2698776

[B75] Suto T. Fukuda M. Ito M. Uehara T. Mikuni M. (2004). Multichannel near-infrared spectroscopy in depression and schizophrenia: cognitive brain activation study. Biol. Psychiatry 55, 501–511. 10.1016/j.biopsych.2003.09.008 15023578

[B76] Tachtsidis I. Elwell C. E. Leung T. S. Lee C.-W. Smith M. Delpy D. T. (2004). Investigation of cerebral haemodynamics by near-infrared spectroscopy in young healthy volunteers reveals posture-dependent spontaneous oscillations. Physiol. Meas. 25, 437. 10.1088/0967-3334/25/2/003 15132309

[B77] Tak S. Ye J. C. (2014). Statistical analysis of fNIRS data: a comprehensive review. Neuroimage 85, 72–91. 10.1016/j.neuroimage.2013.06.016 23774396

[B78] Tomblin J. B. Records N. L. Buckwalter P. Zhang X. Smith E. O'Brien M. (1997). Prevalence of specific language impairment in kindergarten children. J. Speech Lang. Hear. Res. 40, 1245–1260. 9430746 10.1044/jslhr.4006.1245 PMC5075245

[B79] Tsujimoto S. Yamamoto T. Kawaguchi H. Koizumi H. Sawaguchi T. (2004). Prefrontal cortical activation associated with working memory in adults and preschool children: an event-related optical topography study. Cereb. Cortex 14, 703–712. 10.1093/cercor/bhh030 15084489

[B80] Vandenberghe R. Price C. Wise R. Josephs O. Frackowiak R. (1996). Functional anatomy of a common semantic system for words and pictures. Nature 383, 254–256. 8805700 10.1038/383254a0

[B81] Vigneau M. Beaucousin V. Hervé P.-Y. Duffau H. Crivello F. Houde O.. (2006). Meta-analyzing left hemisphere language areas: phonology, semantics, and sentence processing. Neuroimage 30, 1414–1432. 10.1016/j.neuroimage.2005.11.002 16413796

[B82] Villringer A. Dirnagl U. (1994). Coupling of brain activity and cerebral blood flow: basis of functional neuroimaging. Cerebrovasc. Brain Metab. Rev. 7, 240–276. 8519605

[B83] Watson G. S. (1964). Smooth regression analysis, in Sankhyā: The Indian Journal of Statistics, Series A (New Delhi), 359–372.

[B84] Weismer S. E. Plante E. Jones M. Tomblin J. B. (2005). A functional magnetic resonance imaging investigation of verbal working memory in adolescents with specific language impairment. J. Speech Lang. Hear. Res. 48, 405–425. 10.1044/1092-4388(2005/028) 15989401

[B85] Ye J. C. Tak S. Jang K. E. Jung J. Jang J. (2009). NIRS-SPM: statistical parametric mapping for near-infrared spectroscopy. Neuroimage 44, 428–447. 10.1016/j.neuroimage.2008.08.036 18848897

[B86] Young A. E. Germon T. J. Barnett N. J. Manara A. R. Nelson R. J. (2000). Behaviour of near-infrared light in the adult human head: implications for clinical near-infrared spectroscopy. Br. J. Anaesth. 84, 38–42. 10.1093/oxfordjournals.bja.a013379 10740545

